# The promoter activities of sucrose phosphate synthase genes in rice, *OsSPS1* and *OsSPS11*, are controlled by light and circadian clock, but not by sucrose

**DOI:** 10.3389/fpls.2013.00031

**Published:** 2013-03-01

**Authors:** Madoka Yonekura, Naohiro Aoki, Tatsuro Hirose, Kiyoshi Onai, Masahiro Ishiura, Masaki Okamura, Ryu Ohsugi, Chikara Ohto

**Affiliations:** ^1^Bio Research Laboratory, Toyota Motor CorporationToyota, Aichi, Japan; ^2^Laboratory of Crop Science, Graduate School of Agricultural and Life Sciences, The University of TokyoBunkyo-ku, Tokyo, Japan; ^3^NARO Agricultural Research CenterJoetsu, Niigata, Japan; ^4^Center for Gene Research, Nagoya UniversityChikusa-ku, Nagoya, Japan

**Keywords:** sucrose phosphate synthase, promoter–luciferase reporter, transcriptional regulation, circadian clock, sugar sensing, rice

## Abstract

Although sucrose plays a role in sugar sensing and its signaling pathway, little is known about the regulatory mechanisms of the expressions of plant sucrose-related genes. Our previous study on the expression of the sucrose phosphate synthase gene family in rice (*OsSPS*s) suggested the involvement of sucrose sensing and/or circadian rhythm in the transcriptional regulation of *OsSPS*. To examine whether the promoters of *OsSPS*s can be controlled by sugars and circadian clock, we produced transgenic rice plants harboring a promoter–luciferase construct for *OsSPS1* or *OsSPS11* and analyzed the changes in the promoter activities by monitoring bioluminescence from intact transgenic plants in real-time. Transgenic plants fed sucrose, glucose, or mannitol under continuous light conditions showed no changes in bioluminescence intensity; meanwhile, the addition of sucrose increased the concentration of sucrose in the plants, and the mRNA levels of *OsSPS* remained constant. These results suggest that these *OsSPS* promoters may not be regulated by sucrose levels in the tissues. Next, we investigated the changes in the promoter activities under 12-h light/12-h dark cycles and continuous light conditions. Under the light–dark cycle, both *OsSPS1* and *OsSPS11* promoter activities were low in the dark and increased rapidly after the beginning of the light period. When the transgenic rice plants were moved to the continuous light condition, both P_*OsSPS1*_::*LUC* and P_*OsSPS11*_::*LUC *reporter plants exhibited circadian bioluminescence rhythms; bioluminescence peaked during the subjective day with a 27-h period: in the early morning as for *OsSPS1* promoter and midday for *OsSPS11* promoter. These results indicate that these *OsSPS* promoters are controlled by both light illumination and circadian clock and that the regulatory mechanism of promoter activity differs between the two *OsSPS* genes.

## INTRODUCTION

Sucrose is the major photosynthetic product and plays a central role in plant metabolism. Many plant species utilize sucrose as the main sugar for translocation of carbohydrate form source leaves to sink tissues. Sucrose is also reported to be a signal molecule that regulates gene expression in plants (Chiou and Bush, 1998; Vaughn et al., 2002; Ransom-Hodgkins et al., 2003). However, there are fewer reports about sucrose-mediated signaling pathways than glucose-mediated ones (see Rolland et al., 2006 for review).

Sucrose phosphate synthase (SPS, EC 2.3.1.14) catalyzes the conversion of fructose-6-phosphate and UDP-glucose into sucrose-6-phosphate and is known to be the major rate-limiting enzyme in sucrose biosynthesis in plants (Winter and Huber, 2000; Lunn and MacRae, 2003). SPS activity is regulated by allosteric effectors, glucose-6-phosphate (activator) and inorganic phosphate (inhibitor), and by environmental factors such as light and osmotic stress via the phosphorylation of several serine residues. In particular, light–dark modulation of SPS activity via reversible phosphorylation is well established in several plant species (see Winter and Huber, 2000, for review). In addition, the expression of *SPS* genes can be regulated by light and cold stress at the transcriptional level (Chávez-Bárcenas et al., 2000; Lutfiyya et al., 2007; Okamura et al., 2011).

It has been shown that plants have multiple forms of SPS, and that plant *SPS* genes are clustered into four groups (groups A, B, C, and D), based on their amino acid sequences (Castleden et al., 2004; Lutfiyya et al., 2007). Alignment of SPS sequences indicates that all of plant SPS proteins examined possess the phosphorylation site involved in light–dark regulation. The group-D SPS proteins, which can be found only in grass species including rice, are smaller than those from the other groups, lacking two phosphorylation sites involved in 14-3-3 protein binding and osmotic regulation. Rice has five *SPS* genes, *OsSPS*s, classified into four groups: *OsSPS8*, *OsSPS1*, *OsSPS11*, and *OsSPS2 *and *OsSPS6* in groups A, B, C, and D, respectively (Castleden et al., 2004; Okamura et al., 2011). Although each *OsSPS* may have different roles in rice plants, little is known about the physiological significance of each *OsSPS* gene except for *OsSPS1*. For example, the suppression of *OsSPS1* shortens the plant length of rice seedlings (Hirose et al., 2012), and the locus of *OsSPS1* appears to coincide with the quantitative trait locus (QTL) for plant height (Ishimaru et al., 2004). Previously, we measured the mRNA levels of five *OsSPS*s by real-time quantitative RT-PCR with respect to developmental stages, tissues, diurnal changes, and circadian rhythm. We revealed differential expression patterns in the rice *SPS* gene family and part of the complex mechanisms underlying their transcriptional controls (Okamura et al., 2011). We found, in rice leaves, that the expressions of all *OsSPS*s tend to be higher at night than during the day, that *OsSPS1* and *OsSPS6* mRNA levels are negatively correlated with sucrose concentration, and that all *OsSPS*s except for *OsSPS11* exhibit circadian rhythms. These results suggested that the mechanisms of transcriptional control differ between *OsSPS1* and *OsSPS11*; the transcription of *OsSPS1* could be regulated by sugar levels and/or circadian clock and that of *OsSPS11* might not.

In this study, we performed promoter–reporter assays for *OsSPS1* and *OsSPS11* *in vivo* using an automated bioluminescence-monitoring apparatus to investigate the potential regulation of the promoter activities of *OsSPS*s by sugars and/or circadian clock. The promoter activities of transgenic rice plants carrying a promoter–luciferase reporter for *OsSPS1* or *OsSPS11* were measured in real-time under sugar-fed conditions, light–dark cycles, and continuous light. We found that the promoter activities of *OsSPS1* and *OsSPS11* are unaffected by an exogenous supply of sucrose or glucose but are regulated by circadian clock and light.

## MATERIALS AND METHODS

### GROWTH CONDITIONS OF RICE PLANTS AND SAMPLING

Surface-sterilized seeds (*Oryza sativa* L. cv. Nipponbare) were grown for 14 days in Milli-Q water (Millipore, Tokyo, Japan) under 18-h light/6-h dark cycles. The light intensity in the light period was 80 μmol m^-2^ s^-1^ emitted by halogen lamps. The temperature was maintained at 30°C in the light period and 25°C in the dark period. For RNA analysis and the measurement of sugar contents, detached aerial parts of plants were treated with each sugar, immediately frozen in liquid nitrogen, and stored at -80°C.

### P_*SPS1*_::*LUC* AND P_*SPS11*_::*LUC* REPORTER CONSTRUCTS

MultiSite Gateway cloning technology (Life Technologies, Tokyo, Japan) was employed to obtain promoter–reporter constructs containing either *OsSPS1* (Os01g0919400) or *OsSPS11* (Os11g0236100) promoters, modified firefly luciferase gene (*LUC*^+^) as a reporter gene, and nopaline synthase terminator (T_*NOS*_). PCR amplification of the elements required for the constructs was carried out with a high-fidelity DNA polymerase, PrimeSTAR GXL (Takara Bio, Shiga, Japan), according to the manufacturer’s instructions. The primers used to make the constructs are listed in **Table 1**.

**Table 1:** Primer sequences used in this study.

Primer	Nucleotide sequence(5′–3 ′)	Purpose
M13F	GTAAAACGACGG CCAG	Amplification of the Gateway cassettes
M13R	CAGGAAACAGCT ATGAC
OsSPS1-F	GGGGACAACTTTGTATAGAAAAGTTGGATGTGAACCCTGAGCGAGCTTAGATGCATAG	Amplification of P_*OsSPS1*_
OsSPS1-R	GGGGACTGCTTTTTTGTACAAACTTGTCGCCATCTCTCGATCAGCCGATGCTCTC	
OsSPS11-F	GGGGACAACTTTGTATAGAAAAGTTGGACGGACAGCTGCATAGAACGATTAGTCTTTTG	Amplification of P_*OsSPS11*_
OsSPS11-R	GGGGACTGCTTTTTTGTACAAACTTGTCGCCATCCTCCTCCTCCTTCTTCTCC	
LUC-F	GGGGACAAGTTTGTACAAAAAAGCAGGCTCAATGGTCACCGACGCCAAAAACATAAAGAAAGGC	Amplification of *LUC*^+^
LUC-R	GGGGACCACTTTGTACAAGAAAGCTGGGTATTACACGGCGATCTTTCCGCCCTTCTTG	
Tnos-F	GGGGACAGCTTTCTTGTACAAAGTGGGATCGTTCAAACATTTGGCAATAAAG	Amplification of T_*NOS*_
Tnos-R	GGGGACAACTTTGTATAATAAAGTTGCCCGATCTAGTAACATAGATGACAC	
SPS1-QF	TAGCAATGGGAAGCTGGTCT	RT-PCR of *OsSPS1*
SPS1-QR	GATCTGCTCCAGCTTGTTCC	
SPS11-QF	ACCGGAACCTCTACATCGTG	RT-PCR of *OsSPS11*
SPS11-QR	AACTCCACCGCGTACTTCAC	
RUBIQ-F	GGAGCTGCTGCTGTTCTTGG	RT-PCR of *RUBIQ1*
RUBIQ-R	CACAATGAAAACGGGACACGA	

A destination vector, pIG-R4R3, was constructed by introducing a Gateway cassette comprising *att*R4, *ccd*B, chloramphenicol resistance gene (Cm^R^), and *att*R3 into the *Hin*dIII site of an ordinary binary vector, pIG121-Hm (AB489142). Entry clones harboring *SPS* promoters, the reporter, and the terminator were prepared as follows. The promoter regions of *OsSPS1* and *OsSPS11*, P_*SPS1*_ and P_*SPS11*_, respectively, were amplified from genomic DNA from a japonica rice cultivar, Nipponbare, as a template. P_*SPS1*_ and P_*SPS11*_ cover the genome region from -2,415 to +6 and -2,380 to +6 nucleotides from the translation start point, respectively. These two promoter DNAs were cloned into the pDONR P4-P1R vector (Invitrogen, Tokyo, Japan) using BP clonase II (Life Technologies).

A modified gene for firefly luciferase, *LUC*^+^, was amplified from the pSP-*luc*+NF Fusion Vector (Promega, Tokyo, Japan) and cloned into the pDONR221 vector (Life Technologies). Nopaline synthase terminator was amplified from binary vector pBI121 (AF485783; Jefferson et al., 1987) and cloned into the pDONR P2R-P3 vector (Invitrogen).

Finally, these entry clones were assembled into the pIG-R4R3 destination vector using LR Clonase II Plus (Life Technologies) and designated either pSPS1 or pSPS11 vector, in which the initial two amino acid residues of SPSs were fused in frame with luciferase.

### GENERATION OF TRANSGENIC RICE PLANTS

Five-week-old calli derived from a mature rice seed (cv. Nipponbare) were co-cultivated with *Agrobacterium tumefaciens* strain EHA101 harboring either pSPS1 or pSPS11 plasmid for 3 days in N6 (Chu et al., 1975) co-cultivation medium supplemented with 3% (w/v) sucrose, 1% (w/v) glucose, 0.03% (w/v) casamino acids, 2 mg L^-1^ 2,4-dichlorophenoxyacetic acid (2,4-D), 10 mg L^-1^ acetosyringone, and 0.4% (w/v) gelrite at 28°C in the dark. The calli were then transferred to N6 selection medium supplemented with 3% (w/v) sucrose, 1% (w/v) glucose, 0.03% (w/v) casamino acids, 2 mg L^-1^ 2,4-D, 0.4% (w/v) gelrite, 500 mg L^-1^ carbenicillin, and 25 mg L^-1^ hygromycin B as selective antibiotics for 4 weeks (subcultured every 2 weeks) at 30°C under dark conditions. The resistant calli were transferred to MS (Murashige and Skoog, 1962) regeneration medium supplemented with 3% (w/v) sucrose, 3% (w/v) sorbitol, 2% (w/v) casamino acids, 2 mg L^-1^ kinetin, 2 μg L^-1^ 1-naphthylacetic acid, 0.4% (w/v) gelrite, and 25 mg L^-1^ hygromycin B for 4 weeks (subcultured every 2 weeks) to regenerate the aerial parts of rice and then transferred to MS regeneration hormone-free medium for 2 weeks to regenerate the roots at 30°C under continuous light conditions. Regenerated plants (T0 plants) were transplanted into sterilized soil and grown in a greenhouse at 30°C under 12-h light/12-h dark cycles. Self-pollinated heterozygous progenies (T1 seeds and T2 plants) were used for the experiment. Several independent lines were established for P_*OsSPS1*_::*LUC* and P_*OsSPS11*_::*LUC* reporter plants, and all lines exhibited bioluminescence in preliminary assay for luciferase activity. For further experiments, we selected two lines each, the line #8 and #10 as P_*OsSPS1*_::*LUC* reporter plants, and the line #10 and #18 as P_*OsSPS11*_::*LUC* reporter plants, judging from the abundance of seeds.

### MEASUREMENT OF BIOLUMINESCENCE OF P_*OsSPS1*_::*LUC* AND P_*OsSPS11*_::*LUC* TRANSGENIC PLANTS

Plants were grown for 14 days in 100 μM D-luciferin-K (Biosynth, Naperville, IL, USA) dissolved in Milli-Q water under 18-h light/6-h dark cycles, and transferred to continuous light at 30°C. The following day, the aerial parts of each plant were detached, rolled, and transferred to 35-mm petri dishes (BD Biosciences, Tokyo, Japan) containing 3.5 mL of 100 μM D-luciferin. The bioluminescence from each petri dish was subsequently measured using an automated bioluminescence-monitoring apparatus (Okamoto, Onai, and Ishiura, unpublished; model LL04-1; Churitsu Electric Corp., Nagoya, Japan) at 30°C under continuous light or 12-h light/12-h dark cycle conditions. The light intensity was 80 μmol m^-2^ s^-1^ emitted from white fluorescent lamps. To examine the effects of exogenous sugar on the reporter expressions, each sugar was added to the D-luciferin water in each petri dish and the bioluminescence was measured in continuous light condition without resetting the circadian clock. To measure circadian bioluminescence rhythms, each petri dish was exposed to an additional three 12-h light/12-h dark cycles to reset the clock and the bioluminescence was measured in continuous light condition. We analyzed the bioluminescence data using a commercially available bioluminescence-analyzing software (Onai, Shiraki, and Ishiura, unpublished; SL00-01; Churitsu Electric Corp.) based on RAP software (Okamoto et al., 2005). The phase of rhythms was represented as circadian time (CT) calculated by dividing the peak-phase value by the period length and multiplying by 24.

### REAL-TIME QUANTITATIVE RT-PCR

Total RNA was extracted from sugar-treated wild-type (non-transgenic) plants as described by Okamura et al. (2011). Real-time quantitative RT-PCR analysis was performed using sets of gene-specific primers for *OsSPS1* or *OsSPS11* (**Table 1**; Okamura et al., 2011). Total RNA (10 ng) was used for one-step real-time quantitative PCR using the One Step SYBR PrimeScript PLUS RT-PCR Kit (Takara Bio, Shiga, Japan) and SMART Cycler II System (Cepehid Inc., CA, USA) according to the manufacturers’ instructions. The results were normalized according to the transcript levels of a rice polyubiquitin gene, *RUBIQ1* (Wang et al., 2000).

### DETERMINATION OF SOLUBLE SUGAR CONTENT

Soluble sugars were extracted from sugar-treated non-transgenic plants with 80% (v/v) ethanol and determined enzymatically as described by Okamura et al. (2011).

## RESULTS

### EFFECTS OF SUGARS ON THE PROMOTER ACTIVITIES OF *OsSPS1* AND *OsSPS11*

Several independent lines of transgenic rice plants carrying a promoter–luciferase reporter for *OsSPS1* or *OsSPS11* (P_*OsSPS1*_::*LUC* or P_*OsSPS11*_::*LUC*) were established. When D-luciferin was added to the medium, all reporter plants used exhibited bioluminescence and we found no significant differences among lines (data not shown). Therefore, both promoter sequences, P_*OsSPS1*_ and P_*OsSPS11*_, were functional in the rice cells.

**FIGURE 1:**
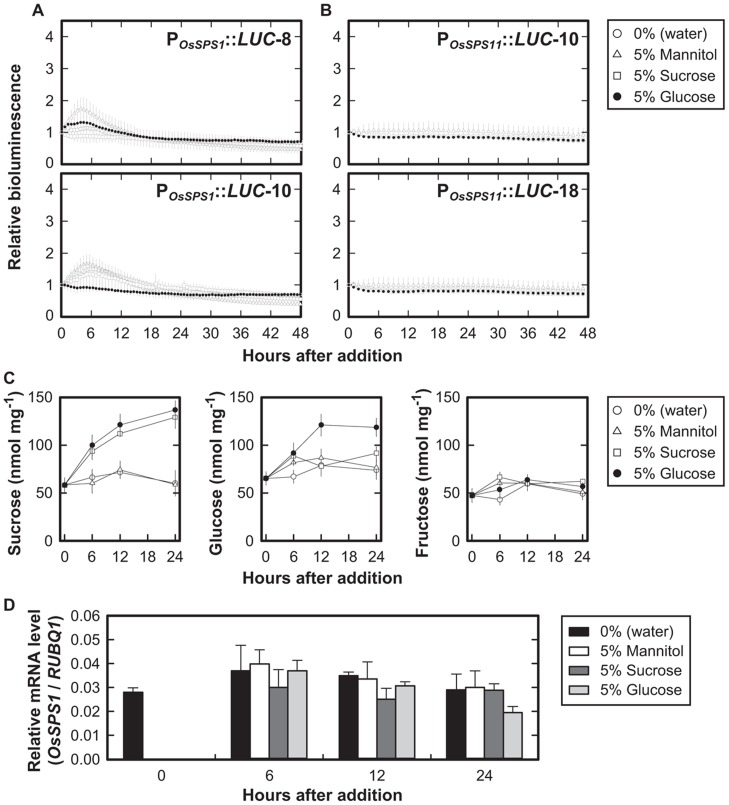
**Promoter activities of *OsSPS1* and *OsSPS11* with the addition of sugars in continuous light**. Fifteen-day-old plants were exposed to sugars added to the medium 24 h after the onset of illumination. **(A)** Bioluminescence of P_*OsSPS1*_::*LUC*****reporter plants without resetting the circadian clock. Data points indicate the mean ±SD of four independent plants in each line. We obtained essentially the same results from three independent experiments. **(B)** Bioluminescence of P_*OsSPS11*_::*LUC*****reporter plants. Data points indicate the mean ±SD of five independent plants in each line. We obtained essentially the same results from three independent experiments. **(C)** Endogenous sugar contents of wild-type (non-transgenic) plants after an exogenous supply of 5% sucrose, 5% glucose, 5% mannitol, or no sugar (0%, water). The *y*-axis represents sugar contents (nmol) per fresh weights of the plants (mg). Error bars indicate the SD of three independent plants. We obtained essentially the same results from three independent experiments. **(D)** mRNA levels of *OsSPS1* in non-transgenic plants after an exogenous supply of 5% sucrose, 5% glucose, 5% mannitol, or water. Values were normalized to the expression level of a rice polyubiquitin gene (*RUBIQ1*). Note that the data at time 0 indicate the relative expression levels before the supply of sugars. Error bars indicate the standard errors of three independent plants. We obtained essentially the same results from three independent experiments.

To examine whether the promoter activities of *OsSPS1* and *OsSPS11 *are regulated by sugars, we measured the bioluminescence of the P_*OsSPS1*_::*LUC* and P_*OsSPS11*_::*LUC* reporter plants after exogenously supplying 5% (w/v) sucrose, 5% (w/v) glucose, or 5% (w/v) mannitol. In both of the reporter plants, bioluminescence levels did not fluctuate significantly during the 48-h continuous light, even without the sugar treatments (0%). We did not observed significant changes in bioluminescence with any treatment (**Figures 1A,B**).

To confirm if exogenous sugars added to the medium were successfully taken up into the plant cells, we examined concentrations of sucrose, glucose, and fructose in the plants after the addition of sugars (**Figure 1C**). When 5% sucrose was added to the medium, the amount of sucrose within the tissues increased 2.5-fold for 24 h whereas glucose and fructose levels remained unchanged. The addition of 5% glucose increased the amount of glucose twofold after 12 h of treatment. The amount of sucrose also increased twofold compared to the control after 6 h of treatment, but fructose levels remained unchanged. No changes in the amounts of these sugars were observed with the addition of 5% mannitol.

We also examined the mRNA levels of *OsSPS1* and *OsSPS11* during the sugar treatments and found no significant differences between *OsSPS1* mRNA levels at any time point (**Figure 1D**). The result was consistent with those from P_*OsSPS1*_::*LUC* reporter plants. In contrast, we did not detect any significant amounts of *OsSPS11* mRNA under these experimental conditions, suggesting low levels of *OsSPS11* mRNA.

These results indicated that sucrose and glucose did not affect the promoter activities of *OsSPS1* or *OsSPS11* even though exogenous sugars in the medium were taken up into the cells.

### DIURNAL CHANGES IN THE PROMOTER ACTIVITIES OF *OsSPS1* AND *OsSPS11*

**FIGURE 2:**
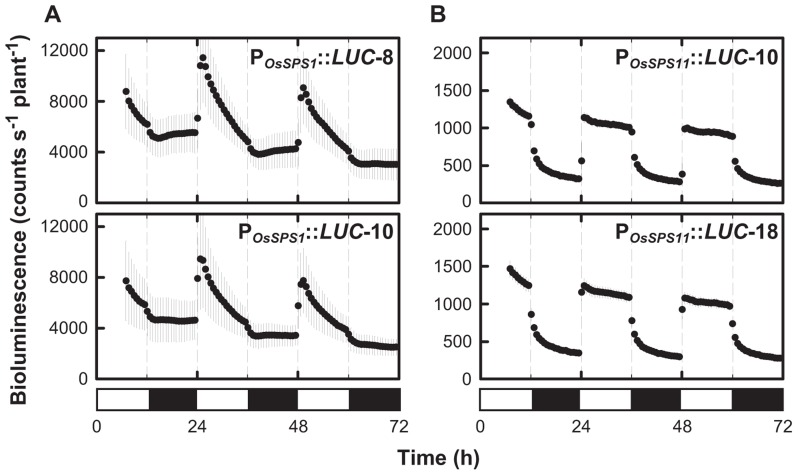
**Diurnal patterns of the promoter activities of *OsSPS1* and *OsSPS11* under light/dark cycles**. The bioluminescence of P_*OsSPS1*_::*LUC* **(A)** and P_*OsSPS11*_::*LUC* **(B)** reporter plants was measured under 12-h light/12-h dark cycles at 30°C.****Data points indicate the mean ±SD from 6 to 8 independent plants in each line. We obtained essentially the same results from three independent experiments. White and black bars represent light and dark periods, respectively.

Previously, we found that *OsSPS1* and *OsSPS11* mRNA levels changed within a day–night cycle (Okamura et al., 2011). We measured the diurnal changes of the promoter activities of *OsSPS1* and *OsSPS11* according to bioluminescence from the reporter plants under 12-h light/12-h dark cycles (**Figure 2**). The promoter activities of both *OsSPS1* and *OsSPS11* were low during the dark period and increased rapidly after the onset of the light period. While the promoter activity of *OsSPS1* decreased gradually during the day and remained low throughout the night (**Figure 2A**), the *OsSPS11* promoter remained active throughout the day and became inactive immediately after the onset of the dark period (**Figure 2B**). These results indicated that the promoter activities of both *OsSPS*s were controlled by light but not in the same manner.

### REGULATION OF THE PROMOTER ACTIVITIES OF *OsSPS1* AND *OsSPS11* BY CIRCADIAN CLOCK

**FIGURE 3:**
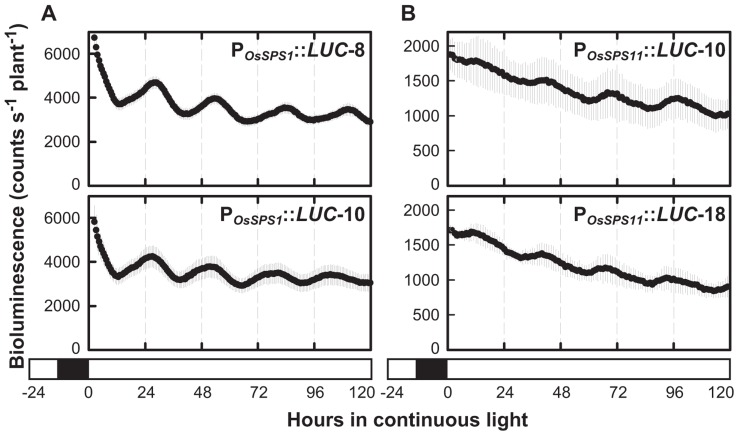
**Bioluminescence rhythms of P_*OsSPS1*_::*LUC***(A)** and P_*OsSPS11*_::*LUC***(B)** reporter plants in continuous light**. Fourteen-day-old plants were exposed to three 12-h light/12-h dark cycles to reset the circadian clock and transferred to continuous light conditions at 30°C for bioluminescence measurement. Data points indicate the mean ±SD from three independent plants in each line. The results are summarized in **Table 2**. We obtained essentially the same results from three independent experiments. White and black bars represent light and dark periods, respectively.

Circadian rhythms are endogenous daily fluctuations in physiological activities sustained under constant conditions with a periodicity of nearly 1 day. As shown in **Figure 1**, neither P_*OsSPS1*_::*LUC* nor P_*OsSPS11*_::*LUC* reporter plants exhibited a significant fluctuation in bioluminescence under continuous light conditions. However, the sugar-feeding experiments were conducted without a pretreatment to synchronize the circadian clock of measured plants, which is a general procedure for measuring circadian rhythms. In fact, when the reporter plants were exposed to three 12-h light/12-h dark cycles prior to the transfer to continuous light conditions, a clear circadian rhythm in bioluminescence was observed in both P_*OsSPS1*_::*LUC* and P_*OsSPS11*_::*LUC *reporter plants (**Figure 3**). The period lengths, peak phases, and amplitudes of rhythms are shown in **Table 2**. Both reporters exhibited circadian bioluminescence rhythms for at least 5 days with period lengths of approximately 27 h. However, these two reporters exhibited different rhythm profiles with respect to their peak phases and amplitudes. The rhythms of the P_*OsSPS1*_::*LUC* reporter plants peaked in the early morning (around CT1), whereas those of P_*OsSPS11*_::*LUC* reporter plants peaked in midday (around CT9). The rhythms of the P_*OsSPS11*_::*LUC* reporter plants were weak; their amplitudes were quarter those of P_*OsSPS1*_::*LUC* reporter plants (0.13 and 0.10 for P_*OsSPS1*_::*LUC* reporter and 0.03 and 0.02 for P_*OsSPS11*_::*LUC* reporter). These results indicated that the promoter activities of both *OsSPS*s were controlled by the circadian clock but their regulations by the clock were distinct.

**Table 2:** Bioluminescence rhythms of P_*OsSPS1*_::*LUC* and P_*OsSPS11*_::*LUC* reporter plants at 30°C under continuous light conditions.

Reporter line	Period length (h)	Phase (CT)	Amplitude	n
P_*OsSPS1*_::*LUC*-8	27.0 ±0.2	1.6 ±0.4	0.13 ±0.02	3
P_*OsSPS1*_::*LUC*-10	25.9 ±0.5	0.9 ±1.0	0.10 ±0.03	3
P_*OsSPS11*_::*LUC*-10	27.4 ±0.8	9.3 ±2.4	0.03 ±0.02	3
P_*OsSPS11*_::*LUC*-18	27.7 ±0.5	8.5 ±1.5	0.02 ±0.01	3

*Values are expressed as the mean ±SD of data obtained in the experiments described in **Figure 3**.*

## DISCUSSION

Since sucrose is a signaling molecule as well as the major photoassimilate in plants, the transcriptional regulation of genes related to the synthesis, transport, and metabolism of sucrose may play roles in sugar sensing and its signaling pathway. However, little is known about the regulatory mechanisms of the expressions of plant sucrose-related genes except for a gene for a proton–sucrose symporter in sugar beet (Vaughn et al., 2002; Ransom-Hodgkins et al., 2003). In the present study, we examined whether genes for SPS can be transcriptionally regulated by sugars in rice plants using promoter–reporter assays for *OsSPS1* and *OsSPS11*. However, neither sucrose nor glucose influenced the activities of the *OsSPS1 *and *OsSPS11* promoters (**Figures 1A,B**). In addition, the sugar treatments did not alter *OsSPS1* mRNA levels in rice plants (**Figure 1D**). These results suggest that the expressions of *OsSPS1* and *OsSPS11* are not regulated by sugar levels in rice plants. There is a possibility that the exogenously supplied sucrose is hydrolyzed by invertases in the apoplast and then taken up into cells in the form of glucose and fructose. If so, it would not be surprising that the effect of sucrose on gene expression looks similar to that of glucose.

It should be noted that feeding sucrose to rice plants induced an increase of sucrose level within the tissues whereas glucose and fructose levels remained unchanged (**Figure 1C**). It is also surprising that glucose feeding induced the accumulation of sucrose as well as glucose. Sucrose feeding in *Arabidopsis thaliana* results in the accumulation of glucose and fructose as well as sucrose (Mita et al., 1997; Sokolov et al., 1998); moreover, sucrose does not accumulate as a result of glucose feeding (Yonekura, Aoki, Hirose, and Onai, unpublished). These differential outcomes of sugar feeding between rice and *Arabidopsis* suggest that caution should be taken when assuming that these plants have the same regulation of sugar metabolism through sugar sensing and signaling.

We also investigated the involvement of light and circadian rhythms in the transcriptional regulation of the two *OsSPS*s. When bioluminescence from *OsSPS1*- and *OsSPS11*-transgenic plants was measured under a light–dark cycle, *OsSPS1 *and *OsSPS11* promoter activities were low in the dark and increased rapidly after the beginning of the light period (**Figure 2**). These results indicate that light is an important cue for the expressions of the two *OsSPS*s at the transcriptional level as well as the post-translational regulation mechanism of SPS protein, which is well-established in several plant species (Winter and Huber, 2000). *OsSPS1* is reported to possess a light-responsive element in its promoter region and that its expression is positively regulated by light (Argüello-Astorga and Herrera-Esstrella, 1996; Chávez-Bárcenas et al., 2000). In the *OsSPS11* promoter sequence, as well as *OsSPS1*, we found putative light-responsive *cis*-acting regulatory elements such as GATA-box and I-box core sequences, which were previously identified in light-regulated genes (Higo et al., 1999; Prestridge, 1991; Terzaghi and Cashmore, 1995). The existence of these *cis*-elements corroborates the present experimental data. However, another interpretation could be given for the diurnal pattern of promoter activities: light illumination may influence indirectly to the promoter activities of *OsSPS1 *and *OsSPS11*. It is generally known in plants that many metabolites fluctuate in response to light–dark transition. The *OsSPS* promoters could be regulated by metabolic changes. Even though sugar levels in tissues did not affect the activities of the two *OsSPS* promoters (**Figure 1**), the sugar levels measured with whole-tissue extracts may not reflect the levels within cells where the *OsSPS*s are able to be active.

The diurnal changes in the promoter activities of *OsSPS1* and *OsSPS11* (**Figure 2**) are inconsistent with our previous data on the day–night accumulation patterns of their mRNAs (Okamura et al., 2011; see Introduction). We can attribute this inconsistency in *OsSPS* expression to post-transcriptional regulation, which influences the stability of mRNAs. For example, in *CIRCADIAN CLOCK ASSOCIATED1* (*CCA1*), one of the clock genes in *Arabidopsis*, the mRNA levels in the P_*CaMV35S*_::*CCA1* plants are constantly high in the dark; meanwhile, in the light, they rapidly decline to about 40% of the dark level. The rapid decrease in *CCA1* transcript level is attributable to the light-modulated stability of the mRNA (Yakir et al., 2007). Likewise, the two *OsSPS* mRNAs might be unstable and degraded immediately in the light, although the transcription of the genes is activated by light. Therefore, it can be speculated that a decrease in the pool size of the transcripts during the daytime enables the sucrose biosynthesis system to respond quickly to rapidly changing environments, including light condition, by the *de novo* transcription of the gene and/or post-translational regulation of the enzyme. In this study, we only used the 5′-untranslated region (UTR). Since Newman et al. (1993) reported that 3′-UTR sequences contribute to mRNA stability like downstream elements, the lack of a 3′-UTR might account for the differences between the diurnal changes in promoter activity and RNA accumulation.

In the sugar-feeding experiments (**Figure 1**), both P_*OsSPS1*_::*LUC* and P_*OsSPS11*_::*LUC* reporter plants did not exhibit circadian rhythms in bioluminescence even under constant condition. However, clear circadian rhythms became detectable when bioluminescence was monitored under constant condition after the reporter plants were exposed to three 12-h light/12-h dark cycles (**Figure 3**). Apparently, the circadian rhythms of promoter activities were masked in the sugar-feeding experiments, probably because the rhythms of four individual plants used were not fully synchronized without the pretreatment before measurements. Although the reason why no circadian rhythms could be detectable without pretreatment still remains unclear, the different results between the two experiments indicate that the pretreatment for synchronization is important to assess the existence of circadian rhythm.

By monitoring bioluminescence in real-time, we found that the decay pattern of promoter activity during the dark period differed between *OsSPS1* and *OsSPS11* (**Figure 2**). We also discovered that both *OsSPS1* and *OsSPS11* promoters are controlled by the circadian clock at least under constant light condition, but their phases and amplitudes were distinct (**Figure 3**). These results clearly show that *OsSPS1* and *OsSPS11* are regulated differently at the transcriptional level, implying multiplicity in the transcriptional control mechanisms of the five *OsSPS*s. Further studies at the both transcriptional and post-transcriptional levels are necessary to fully elucidate the complex regulatory mechanisms of the expressions of *OsSPS*s and characterize the roles of the five *OsSPS*s in sucrose metabolism and assimilate partitioning in rice plants.

## Conflict of Interest Statement

The authors declare that the research was conducted in the absence of any commercial or financial relationships that could be construed as a potential conflict of interest.
